# Active hydrodynamic imaging of a rigid spherical particle

**DOI:** 10.1038/s41598-020-58880-0

**Published:** 2020-02-14

**Authors:** Daisuke Takagi, J. Rudi Strickler

**Affiliations:** 1 Department of Mathematics, University of Hawaii at Manoa, Honolulu, HI 96822 USA; 2 Pacific Biosciences Research Center, University of Hawaii at Manoa, Honolulu, HI 96822 USA; 3 Department of Biological Sciences, University of Wisconsin – Milwaukee, Milwaukee, WI 53204 USA; 4 Marine Science Institute, University of Texas at Austin, Port Aransas, TX 78373 USA

**Keywords:** Fluid dynamics, Biological physics

## Abstract

A body with mechanical sensors may remotely detect particles suspended in the surrounding fluid via controlled agitation. Here we propose a sensory mode that relies on generating unsteady flow and sensing particle-induced distortions in the flow field. We demonstrate the basic physical principle in a simple analytical model, which consists of a small spherical particle at some distance from a plate undergoing impulsive or oscillatory motion. The model shows that changes in pressure or shear on the plate can be used to infer the location and size of the sphere. The key ingredient is to produce strong shear or strain around the sphere, which requires careful tuning of the viscous boundary layer on the moving plate. This elucidates how some organisms and devices may control their unsteady dynamics to enhance their range of perception.

## Introduction

Living organisms can display highly unsteady behaviour, for instance when they are searching and hunting for food. For example, blind cavefish swim in a series of bursts with sudden changes in velocity^1^, while copepods vigorously vibrate their appendages^2^. The dynamics of the animal body drives the surrounding fluid to flow, and the effects on locomotion and feeding are particularly well documented^3,4^. What remains less clear is to what extent the unsteady flow helps in sensing particles suspended in the vicinity. This inspires fundamental problems in physics with potential applications in engineering sensors.

Sensing objects remotely based on disturbances generated in the flow field is commonly known as hydrodynamic imaging. The disturbances may manifest as mechanical stress on the surface of an organism equipped with flow sensors, such as the array of hair-like neuromasts on fish^5^ and mechanosensitive hairs on arthropods^6^, which include copepods^7^. A large object moving quickly past the sensors could be readily detected^8^, as modelled using potential theory for inviscid flow^9–12^. However, a small particle can generate only weak disturbances by itself, e.g., due to buoyancy^13,14^, deformation or/and propulsion^15–18^. A neutrally buoyant and inactive particle, hereafter referred to as an inert particle, remains stationary in a quiescent fluid or remains entrained in fluid flow with little or no additional disturbance, rendering it difficult to detect passively.

Here we show that such an inert particle can be detected *actively* by a body that adequately agitates the fluid environment. The key ingredient is to produce strong shear or strain around the rigid particle, which in turn induces distortions in the flow field and becomes detectable from a distance. Contrary to common belief, the particle’s deformation or translation relative to the sensors can be secondary and unnecessary for detection. Our study builds on two previous studies concerned with the detection of an inert particle suspended in slow viscous flows^19,20^. They demonstrated that the difference between fluids with and without a suspended particle could be distinguished based on the modified flow field, though they did not resolve how the particle’s location and size could be determined. Moreover, previous models of hydrodynamic imaging assumed the flow to be steady or inertialess in the viscous Stokes limit, a physical regime where the fluid responds instantaneously and retains no memory of the past. Our study considers the problem of locating an inert particle in unsteady as opposed to steady flow and elucidates how the effects of fluid viscosity and inertia can be carefully tuned to enhance the sensing performance.

To demonstrate the basic physical principle of locating a small particle in a viscous flow field with inertia, we develop a simple proof-of-concept model consisting of a rigid spherical particle near a moving plate. The plate is assumed to undergo impulsive or oscillatory motion, which is partly inspired by the transient bursts of blind cavefish^1^ and the periodic beats of copepods^2^, though the surface of the animals’ body is crudely approximated by a flat and infinitely long plate in the model. The problem with a body of finite size is that, if inertial and viscous effects are included, then even a shape as simple as a sphere generates complex flows that are difficult to solve analytically. Instead of resorting to numerical simulations with specific parameter values, we consider a simpler model to obtain analytical results, which are arguably easier to inspect and interpret in general. The results may not apply to any flow field around moving organisms in quantitative detail but they still help in elucidating the fundamental physics of detecting particles remotely through careful agitation. The theory essentially extends the classical Stokes’ problems for impulsive and oscillatory flows^21^ by introducing a rigid particle in the fluid. We anticipate that the simplified model will serve a few different purposes in the future: as a foundation for developing improved models of hydrodynamic imaging in more complex flow fields; as a tool for broadly assessing what organisms are capable of sensing mechanically; and as a source of inspiration for engineering novel imaging devices in practical applications.

## Results

### Mathematical model of an impulsive plate

**Figure 1:**
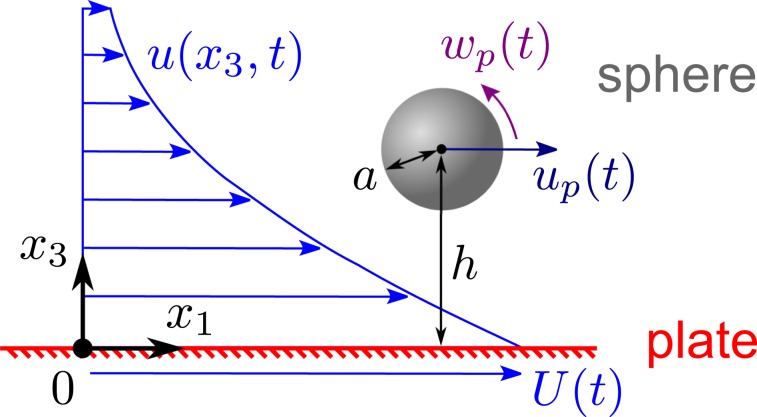
Sketch of a plate translating, generating flow, and driving a sphere to move.

We first consider flow generated by a rigid plate moving instantaneously from rest, a classical problem^21^ known as the Stokes’ first problem as commonly featured in introductory books on fluid dynamics^22^. Suppose the plate lies in the $({x}_{1},{x}_{2})$ plane and is surrounded by an incompressible Newtonian fluid of density ${\rho }_{f}$, dynamic viscosity $\mu$ and kinematic viscosity $\nu =\mu /{\rho }_{f}$. The fluid and the plate are stationary until time $t=0$, when the plate starts translating at constant speed $U > 0$ along the ${x}_{1}$ axis (Fig. 1). At subsequent times $t > 0$, the fluid is dragged along the ${x}_{1}$ direction with speed

1 $$u({x}_{3},t)=U(1-\frac{2}{\sqrt{\pi }}{\int }_{0}^{\frac{{x}_{3}}{2\sqrt{\nu t}}}{e}^{-{r}^{2}}dr)=U{\rm{e}}{\rm{r}}{\rm{f}}{\rm{c}}(\frac{{x}_{3}}{2\sqrt{\nu t}}),$$

which decays with distance ${x}_{3}\ge 0$ from the plate (Fig. 1). This flow field is an exact solution of the governing Navier-Stokes equations and is valid for *any* plate speed *U* as long as the flow remains laminar without turbulence. In the absence of particles, the plate experiences uniform pressure and shear stress

2 $${\sigma }_{0}=\mu {\frac{\partial u}{\partial {x}_{3}}|}_{{x}_{3}=0}=-\,\frac{\mu U}{\sqrt{\pi \nu t}}.$$

We extend this problem by accounting for perturbations due to a small spherical particle.

We introduce a rigid sphere of radius *a* centered at a distance *h* from the plate (Fig. 1). The analysis of the motion of the sphere is greatly simplified if the parameter $\alpha =a/h$ is sufficiently small as assumed in our model for the following reasons. The sphere translates almost with the fluid flow predicted by Eq. (1), provided that the local flow relative to the moving sphere has negligible inertia. To illustrate when this occurs, we assume for simplicity that the sphere’s density ${\rho }_{s}$ is not much greater than the fluid density ${\rho }_{f}$. In the reference frame of the moving sphere, the fluid immediately surrounding it is driven predominantly by spatial gradients in the background flow, and this is assumed to have small shear Reynolds number^23^

3 $$\frac{\alpha {\rho }_{f}Ua}{\mu }\ll 1.$$

This expression is obtained with the characteristic length scale set by *a* and the characteristic time scale set by $h/U$, the inverse of the typical fluid velocity gradient. Physically, this means that, while the fluid and sphere may both have inertia, the local fluid flow relative to the sphere is governed predominantly by viscosity, not inertia. In this viscous regime, the motion of the sphere can be readily predicted using Faxen’s laws (see Appendix), assuming the background flow far from the sphere is given by Eq. (1). This assumption is valid for a sphere sufficiently far from the plate in the limit of small $\alpha \ll 1$.

### Particle motion and strain

The transient dynamics of the sphere is conveniently described in terms of dimensionless time $\tau =t/T$, where $T={h}^{2}/\nu$ is the typical time needed for the growing boundary layer on the plate to reach the sphere. The sphere is predicted to translate with speed ${u}_{p}$ in the ${x}_{1}$ direction according to

4 $$\frac{{u}_{p}}{U}={\rm{erfc}}(\frac{1}{2\sqrt{\tau }})+\frac{{\alpha }^{2}}{12\sqrt{\pi {\tau }^{3}}}{e}^{-\frac{1}{4\tau }},$$

which is obtained from Faxen’s first law for a force-free sphere. The total force on the sphere is negligible provided that the condition (3) holds and that effects due to external forces such as gravity are insignificant as discussed later. Similarly, the total torque on the sphere is neglected. The plate-induced shear in the surrounding fluid causes the sphere to rotate around the ${x}_{2}$ axis with angular speed ${w}_{p}$ given by

5 $$\frac{{w}_{p}}{U/h}=\frac{1}{2\sqrt{\pi \tau }}{e}^{-\frac{1}{4\tau }},$$

which is obtained from Faxen’s second law for a torque-free sphere. The typical translation and rotation of the sphere are animated in Movie S1.

**Figure 2:**
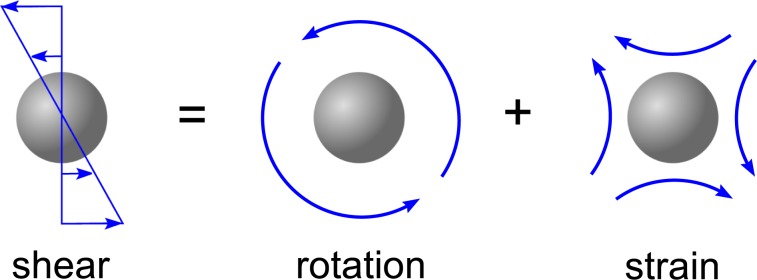
Shear flow in the vicinity of the translating sphere can be interpreted as the sum of rotating and straining flows.

The rotation of the sphere has little effect on the far-field disturbance, which is more importantly influenced by the sphere’s rigidity and resistance to strain. The disturbance is illustrated by moving in the reference frame of the translating sphere, where the background shear flow can be interpreted as a sum of rotational and straining flows as sketched in Fig. 2. The straining flow exerts stress on the rigid sphere, which exerts an equal and opposite stress on the fluid. The resultant stress is perceived in the far-field as a force dipole also known as a stresslet. The strength *S* of the stresslet is given by

6 $$\frac{S}{\mu U{a}^{2}}=\frac{10\sqrt{\pi }}{3}\frac{\alpha }{\sqrt{\tau }}(1+\frac{{\alpha }^{2}}{40{\tau }^{2}}(1-2\tau )){e}^{-\frac{1}{4\tau }},$$

which depends on the degree of strain around the sphere according to Faxen’s third law (see Appendix). A similar result is expected if the rigid sphere is replaced by a liquid droplet or gas bubble, provided that it remains approximately spherical with sufficient viscosity or surface tension. But a non-spherical particle would experience different strain rates depending on its orientation^24^. We restrict our attention to a rigid sphere so that its rotation can be neglected for simplicity in the remaining analysis.

### Stress on the plate

The stresslet centred at the sphere importantly perturbs the far-field flow and modifies the stress on the plate. The stresslet produces a radial flow pattern but this violates the no-slip condition on the plate. To reestablish this condition, we construct a suitable image system consisting of fundamental singularities of Stokes flow (see Appendix). Thus, we account for the presence of the plate in our predictions for the sphere-induced stress presented below.

**Figure 3:**
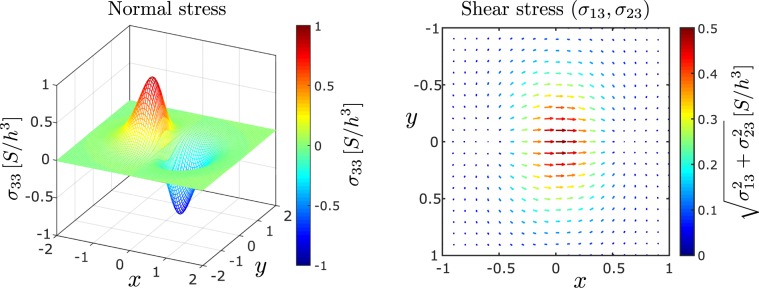
Normal and shear stresses on the plate centred at the point closest to the sphere.

We introduce rescaled coordinates $x=({x}_{1}-{x}_{p})/h$ and $y={x}_{2}/h$ originating from the point on the plate closest to the particle. In terms of these new coordinates, the normal stress on the plate is given by (see Appendix)

7 $${\sigma }_{33}=-\,p(x,y)=\frac{3S}{\pi {h}^{3}}(\frac{1}{{\rho }^{5}}-\frac{5}{{\rho }^{7}})x,$$

where $\rho =\sqrt{{x}^{2}+{y}^{2}+1}$ denotes the rescaled distance from the particle. Figure 3 shows that the disturbance creates regions of high and low pressure, respectively, ahead and behind the closest point to the particle moving on the plate. A typical pressure profile on an impulsive plate along the *x*_1_ axis is animated in Movie S1.

The shear stress on the plate across the flow is given by (see Appendix)

8 $${\sigma }_{23}=\mu {\frac{\partial {u}_{2}}{\partial {x}_{3}}|}_{{x}_{3}=0}=\frac{3S}{2\pi {h}^{3}}(\frac{1}{{\rho }^{5}}-\frac{5}{{\rho }^{7}})xy,$$

while the shear stress on the plate along the flow is given by ${\sigma }_{0}+{\sigma }_{13}$, where

9 $${\sigma }_{13}=\mu {\frac{\partial {u}_{1}}{\partial {x}_{3}}|}_{{x}_{3}=0}=\frac{3S}{2\pi {h}^{3}}(\frac{1+{x}^{2}}{{\rho }^{5}}-\frac{5{x}^{2}}{{\rho }^{7}}).$$

The shear stresses are represented by arrows in Fig. 3. For small $\rho \ge 1$, the arrows have a positive $x$ component contrary to ${\sigma }_{0}$, indicating that the sphere-induced shear reduces the original shear on the plate.

The shear and normal stresses peak at different locations. By setting $\nabla {\sigma }_{i3}=0$, we find that the shear stress peaks at the point on the plate closest to the particle, while the normal stress peaks in magnitude immediately ahead and behind the closest point at $x=\pm \,\sqrt{(27-\sqrt{665})/8}\approx \pm \,0.389$. The maximum change in stress is of the form $\hat{k}S/{h}^{3}$, where $\hat{k}=3/2\pi$ for maximum shear or $\hat{k}\approx 0.873$ for maximum pressure. The plate experiences a larger change in pressure than in shear at any given time.

**Figure 4:**
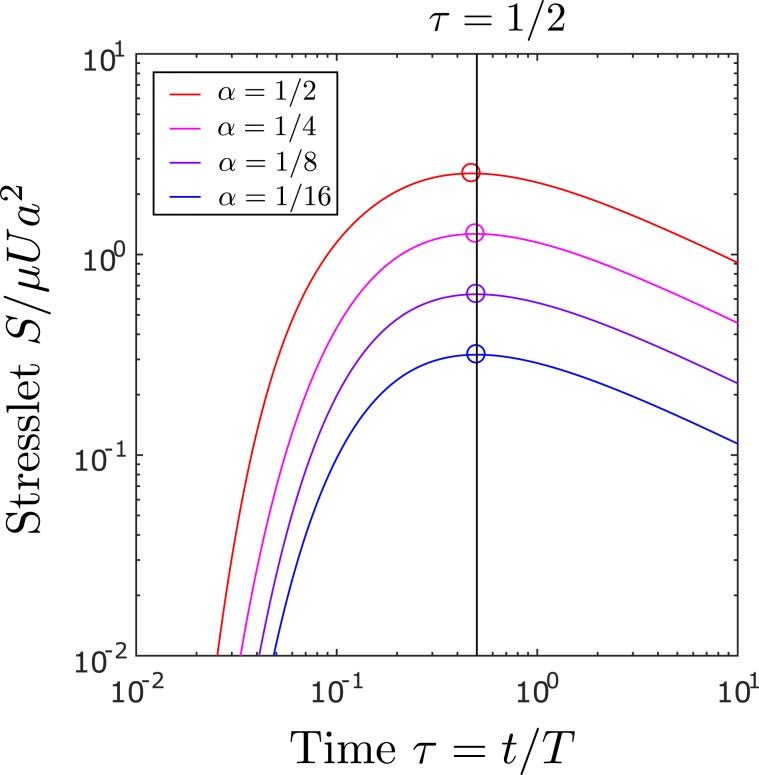
Stresslet signal from a sphere rises then decays over time $\tau$ following an impulsive motion of the plate. Circles show peaks which are expected to occur at $\tau =1/2$ in the limit as $\alpha \to 0$ according to Eq. (6).

The stress on the plate is proportional to the stresslet strength *S* in Eq. (6), which evolves over time as plotted in Fig. 4. In the limit as $\alpha \to 0$, the strength *S* peaks when the particle is sheared most by the boundary layer at $\tau =1/2$. Evaluating *S* at $\tau =1/2$, we obtain the maximum change in stress

10 $${\sigma }_{m}=\bar{k}{\alpha }^{3}\frac{\mu U}{h},$$

where $\bar{k}=5\sqrt{2/e\pi }$ for maximum shear or $\bar{k}\approx 4.42$ for maximum pressure. While the magnitude of the original shear $|{\sigma }_{0}|$ decays over time and the perturbed shear ${\sigma }_{13}$ also decays for $\tau  > 1/2$, the ratio ${\sigma }_{13}/|{\sigma }_{0}|$ increases monotonically and approaches $5{\alpha }^{3}$ in the long-time limit.

### Oscillating plate

We now consider the plate to oscillate sinusoidally along the ${x}_{1}$ axis with amplitude *A* and angular frequency $\omega$, another classical problem^21^ known as the Stokes’ second problem. The plate oscillates with velocity $V\,\cos \,\omega t$, where $V=A\omega$ is the maximum speed. The resultant fluid flow velocity is given by

11 $$\tilde{u}({x}_{3},t)=V{e}^{-{x}_{3}/\delta }\,\cos (\omega t-\frac{{x}_{3}}{\delta }),$$

where $\delta =\sqrt{2\nu /\omega }$ is the Stokes boundary layer thickness. In the absence of particles, the plate experiences uniform pressure again, but the shear stress is now given by

12 $${\tilde{\sigma }}_{0}=-\,\sqrt{2}\frac{\mu V}{\delta }\,\cos (\omega t+\frac{\pi }{4}).$$

The effects of a rigid sphere with small parameter $\alpha =a/h$ can be predicted with the same procedure as before, using Eqs. (11 and 12) instead of Eqs. (1 and 2). We introduce another dimensionless parameter $\eta =h/\delta$, the distance rescaled by the boundary layer thickness. The condition in (3) about small Reynolds number is modified by

13 $$\frac{\alpha {\rho }_{f}Va}{\mu }\eta {e}^{-\eta }\ll 1.$$

In this case, the sphere is predicted to move with translational velocity ${\tilde{u}}_{p}$, angular velocity ${\tilde{w}}_{p}$, and experience strain producing a stresslet of strength $\tilde{S}$ according to

14 $$\frac{{\tilde{u}}_{p}}{V}={e}^{-\eta }\sqrt{1+\frac{{(\alpha \eta )}^{4}}{9}}\,\cos (\omega t-\eta +{\tan }^{-1}\frac{{(\alpha \eta )}^{2}}{3}),$$

15 $$\frac{{\tilde{w}}_{p}}{V/\delta }=\frac{1}{\sqrt{2}}{e}^{-\eta }\,\cos (\omega t-\eta +\frac{\pi }{4}),$$

16 $$\frac{\tilde{S}}{\mu V{a}^{2}}=R\,\cos (\omega t-\eta +{\tan }^{-1}\frac{5+{(\alpha \eta )}^{2}}{5-{(\alpha \eta )}^{2}}),$$

where

17 $$R=\frac{10\sqrt{2}\pi }{3}\alpha \eta {e}^{-\eta }\sqrt{1+\frac{{(\alpha \eta )}^{4}}{25}}$$

is the dimensionless amplitude of the oscillating stresslet. Oscillations in ${\tilde{u}}_{p}$, ${\tilde{w}}_{p}$, and $\tilde{S}$ all have the same angular frequency $\omega$ but different amplitudes and phase delays in general. Note that $\tilde{S}$ and ${\tilde{\sigma }}_{0}$ can have the same sign for some duration of time, meaning that the sphere-induced shear can reinforce the original shear on the oscillatory plate, contrary to the reduced shear on the impulsive plate as presented earlier.

**Figure 5:**
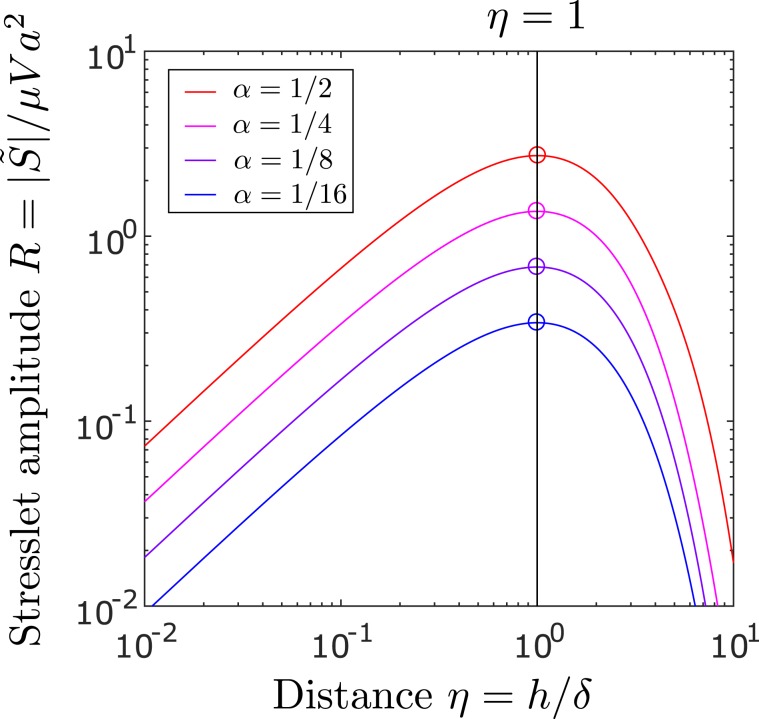
Amplitude of stresslet oscillation from a sphere depending on its distance from an oscillatory plate. Circles show peaks, which are expected at $\eta =h/\delta =1$ in the limit as $\alpha \eta \to 0$ according to Eq. (17).

The sphere-induced stress is again given by Eqs. (7–9) but with stresslet strength *S* replaced by $\tilde{S}$, which depends importantly on the angular frequency $\omega$ of the oscillatory plate. As $\omega$ increases, the boundary layer thickness $\delta =\sqrt{2\nu /\omega }$ decreases, which increases $\eta =h/\delta$ for a fixed particle distance *h*. A moderate value of $\eta$ maximises the stresslet amplitude *R* as plotted in Fig. 5. Physically, the particle is most sheared when the boundary layer has thickness comparable to the particle-plate distance at $\eta \approx 1$. This corresponds to a maximum change in stress

18 $${\tilde{\sigma }}_{m}=\tilde{k}{\alpha }^{3}\frac{\mu V}{h},$$

where $\tilde{k}=5\sqrt{2}/e$ for maximum shear or $\tilde{k}\approx 4.76$ for maximum pressure on an oscillatory plate. Note that $\tilde{k}=\bar{k}\sqrt{\pi /e}$ is slightly larger than $\bar{k}$ in Eq. (10), indicating that an oscillatory plate can experience a little more disturbance in stress than an impulsive plate moving at the same maximum speed $U=V$. We note that, as the frequency $\omega$ or the parameter $\eta$ decreases and approaches 0, the particle-induced shear and the original shear on an oscillating plate both decrease in amplitude, but the ratio of the two amplitudes increases and approaches $5{\alpha }^{3}$, the same limit as the long-term ratio of the particle-induced and original shear stresses computed earlier on an impulsive plate. A typical pressure profile on an oscillatory plate along the ${x}_{1}$ axis is animated in Movie S2.

## Discussion

We discuss the physical implications and limitations of our model first, followed by some possible biological interpretations further below. The model shows that a sphere suspended in a fluid can be imaged in theory by sensing pressure or shear on a moving plate. At any instant, the profile in normal or shear force (Fig. 3) determines the point on the plate closest to the sphere. By tracking the closest point over time, the translational velocity of the sphere can be measured, which enables ranging, that is, determining the approximate distance *h* to the sphere using Eqs. (4) or (14). The effect of the small parameter *α* on the translational velocity could be neglected to a first approximation. Once *h* is estimated, the radius *a* of the sphere can be estimated by measuring stress changes, which scale like *a*^3^ according to Eqs. (6) or (16). Thus the stress on the plate decodes the sphere’s location and size.

The active sensing mechanism described above has the advantage that the stress changes on the plate are proportional to the maximum plate speed, which can be controlled and enhanced as needed. This would not be possible with a passive sensing mechanism that relies on the target particle to generate a sufficient signal by itself. To illustrate this further, we consider a buoyant or sinking sphere with a density difference $\Delta \rho$ from the fluid density. The sphere exerts a gravitational force $F \sim \Delta \rho g{a}^{3}$ on the fluid, which is expected to produce stress changes on the order of $\Delta \rho gh{\alpha }^{3}$ on the plate^25,26^, regardless of the plate speed. If the signal due to gravity is sufficiently strong then it could be detected passively on a stationary plate. Otherwise, the particle must be detected actively by moving the plate at sufficient speeds to induce a much stronger signal. This arises on an impulsive or oscillatory plate when

19 $$\frac{\Delta \rho g{h}^{2}}{\mu U}\ll 1,\,\frac{\Delta \rho g{h}^{2}}{\mu V}\ll 1,$$

conditions independent of the sphere radius *a*. The dimensionless numbers on the left are useful measures of the relative potential for passive and active sensing. Under the conditions in (19), the particle experiences negligible effects due to gravity and is more importantly detected by the dynamics of the plate as described in our model.

The proposed mechanism is suited particularly for the rapid sensing of particles suspended in close proximity. The predicted signal peaks when the distance to the particle becomes comparable to the thickness of the viscous boundary layer on the plate, which occurs when the time elapsed from an instant start or the period of oscillation is of order ${h}^{2}/2\nu$. This time scale becomes intolerably long at distances *h* beyond several centimetres. But for *h ~* 1 mm, the time scale is less than a second in air or water. For comparison, it is orders of magnitude lower than the characteristic time for chemical diffusion, ${h}^{2}/D$, where the rate of molecular diffusion *D* is typically much smaller than the rate of viscous dissipation $\nu$. Thus, particles could be located remotely with higher precision by relying on fast mechanical signals as described here instead of slow chemical diffusion.

In order to obtain an image with adequate resolution, the plate must be equipped with sensors that can detect the predicted stress variations in time and space. Let ${\sigma }_{s}$ denote the smallest change in stress that is detectable by the sensors. The critical distance ${h}^{\ast }$ from an impulsive plate beyond which the sphere becomes undetectable is given by

20 $${h}^{\ast }={(\bar{k}\mu U{a}^{3}/{\sigma }_{s})}^{1/4},$$

which is obtained by rearranging Eq. (10). A similar relation

21 $${h}^{\ast }={(\tilde{k}\mu V{a}^{3}/{\sigma }_{s})}^{1/4}$$

is obtained from Eq. (18) for the critical distance from an oscillatory plate. In either case, ${h}^{\ast }$ scales like ${a}^{3/4}$. There is a relatively weak dependence of ${h}^{\ast }$ on the other parameters, given that a 16-fold change in ${\sigma }_{s}$, *U* or *V* causes only a 2-fold change in ${h}^{\ast }$. We discuss further below whether and to what extent the mechanical sensors on animals and potentially machines can sense the small stress changes predicted in our model.

A major limitation of the current model lies in the assumption that the particle is driven by a flat and infinitely-long plate, resulting in the surrounding fluid flowing parallel to the plate. The flow may remain approximately parallel near a slightly curved and finite-sized body, provided that the distance from the particle to the closest part of the body is much smaller than the body’s local radius of curvature, as well as the distance to the ends of the body. The particle dynamics is expected to be governed locally by the viscous boundary layer, similar in dynamics as predicted in our model as long as the boundary layer remains thin and adhered to the body. But in our model, the boundary layer on an impulsive plate grows indefinitely and eventually occupies the entire fluid region, which does not necessarily develop around a finite-sized body. Depending on the size, shape and speed of the body, the boundary layer may detach and shed vortices into its wake. This could profoundly modify the flow field and limit the scope of our model. Nevertheless, at sufficiently early times, the boundary layer is expected to grow with time in a similar manner as near an infinite plate on minimally curved parts of the body. Similarly, on a finite body undergoing oscillatory motion, the boundary layer thickness could decrease with increasing frequency, at least qualitatively like the Stokes boundary layer on an infinite plate. Hence the concept of actively controlling the boundary layer to detect particle suspensions may offer useful qualitative insights into some biological behaviour.

We discuss several possible interpretations of the behaviour of fish and copepods. These animals generate highly unsteady flows much richer in complexity than those considered in our model. Nonetheless, the model still provides an order-of-magnitude estimate of the expected mechanical stresses on the body, based on the characteristic length and time scales of the unsteady motion of the animals. This offers the opportunity to compare with the reported sensitivity of their sensors and assess whether the proposed mechanism is at least feasible in theory. The comparison should only be treated as indicative because our model does not fully account for the dynamics of the animals and their surroundings in quantitative detail.

A common swimming behaviour of fish consists of the body bursting into motion and then gliding for a relatively long duration^1^. This unsteady swimming behaviour has been interpreted as minimising energetic costs^27^ and signal noise such as vision blur^28^. Copepods show similar unsteady dynamics by abruptly jumping then gradually slowing down over time, which has been interpreted as stealthy^29^. Here we offer an additional, radically different interpretation that the unsteadiness is suited for probing and detecting particles suspended in the vicinity. The detection does not require any self-generated disturbance of the particles, e.g. due to vibrations^30^, because even the inert particles are detectable as shown here, particularly at the edge of the viscous boundary layer that grows with time on an impulsively started plate. Thus the particles at different distances from a surface bursting into motion could be effectively scanned over time.

**Figure 6:**
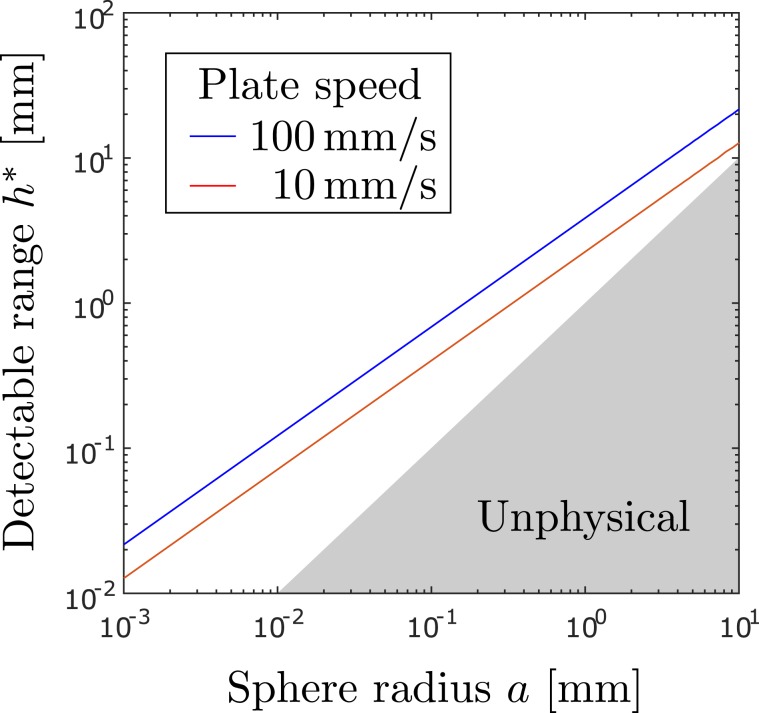
A sphere of radius *a* becomes undetectable beyond a critical distance ${h}^{\ast } \sim {a}^{3/4}$. Two representative curves are shown for the theoretical limit on perception on a plate bursting into motion at speed *U* = 100 mm/s and oscillating with maximum speed *V* = 10 mm/s. The curves are given by Eqs. (20) and (21) with stress sensitivity ${\sigma }_{s}=2\times {10}^{-3}$ Pa s and water viscosity $\mu ={10}^{-3}$ Pa s.

We explore this feasibility by comparing the expected stress changes on a swimming fish and the reported sensitivity of the flow sensors. The lateral line system of fish is reported to be able to detect pressure gradients of ${\sigma }_{s} \sim 2\times {10}^{-3}$ Pa across a distance of 2 mm on the surface of the body^5^. Such a sensory surface moving impulsively with characteristic speed $U \sim 100$ mm/s through water is capable of detecting spheres at a range of distances according to Eq. (20). The dependence of the detectable range on the sphere radius *a* is plotted in Fig. 6. The largest detectable distance is expected to be on the order of a few centimetres, given that ${h}^{\ast } \sim a \sim \bar{k}\mu U/{\sigma }_{s}$ at that scale. This still allows a wide range of particle sizes to be detectable around swimming fish in theory. For example, if a blind cavefish bursts into motion from rest and continues gliding at speed *U* ~ 100 mm/s^1^, a particle of radius *a* ~ 0.5 mm at a distance *h* ~ 2 mm from the fish surface becomes detectable after a typical time on the order of a second, when the pressure changes on the swimming fish are expected to reach a level on the order of $3\times {10}^{-3}\,\text{Pa} > {\sigma }_{s}$. The condition in (19) is satisfied for particle densities with $\Delta \rho  \sim {10}^{-2}{\rho }_{f}$, meaning that such particles are likely to be detected actively by the swimming fish.

We next turn our attention to the back-and-forth motion of appendages while copepods feed on microscopic prey. It was identified long ago that the oscillatory nature of the flow^31^ and viscous effects near the appendages^2^ must play an important role in detecting nearby objects. Numerous types of models have been developed for the flow field around feeding copepods^32^. However, previous studies of sensing assumed either oscillatory inviscid flow or steady viscous flow in the far field^14,20,33^ and neglected the combined effects of viscosity and inertia in the near field. More recent studies have shown that, even in fluids dominated by viscosity at the microscopic scale, oscillating structures such as cilia have minute inertia yet they can still have profound long-lasting effects, resulting in flows governed importantly by both viscosity and inertia^34,35^. It is precisely the combination of these two effects that can effectively facilitate the detection of inert particles at the edge of a boundary layer, e.g. on an oscillating plate as shown here. The boundary layer thickness depends on the frequency of oscillation so this could be simply adjusted to scan for particles at different distances. This offers valuable insight into why copepods can handle and locate food particles in the near field without touching them^36^.

We explore the feasibility of this again by comparing the expected stress changes on the surface of a vibrating appendage and the reported sensitivity of mechanosensory hairs of copepods. The hairs have been reported to detect flow speeds $v \sim 20\,\mu {\rm{m}}/{\rm{s}}$^7^. This corresponds to a threshold shear of ${\sigma }_{s} \sim \mu v/l \sim 2\times {10}^{-3}$ Pa, taking $l \sim 20\,\mu {\rm{m}}$ as the typical hair length and assuming that the hairs deflect linearly to small shear^37^. Incidentally, a similar threshold shear is obtained for artificial sensors with $l \sim 200\,\mu {\rm{m}}$ and $v \sim 200\,\mu m/s$^38^. We consider the detectable range of such hairs mounted on an oscillatory plate with $V \sim 10\,\text{mm}/s$, the typical peak speed of appendages recorded in the feeding behaviour of copepods^2^. Assuming the hairs are distributed on the appendages, they may be able to detect prey with a range of microscopic sizes according to Eq. (21) and Fig. 6. For example, if a spherical algal cell of radius $a \sim 20\,\mu {\rm{m}}$ is at a distance $h \sim 100\,\mu {\rm{m}}$ from an appendage vibrating at a frequency $\omega  \sim 100\,/{\rm{s}}$, the cell is predicted to induce shear changes on the order of $2\times {10}^{-3}$ Pa, which is detectable in theory. For a cell with density difference $\Delta \rho  \sim {10}^{-2}{\rho }_{f}$ we obtain $\Delta \rho g{h}^{2}/\mu V \sim {10}^{-1}\ll 1$, meaning that the detection is influenced less by gravity and more importantly by the vibrations of appendages based on the physical principle explained here. The current model of active hydrodynamic imaging may be extended in the future by incorporating fluid-structure interactions and accounting for the shape and dynamics of the sensing structure in more realistic flow fields.

In conclusion, inert particles suspended in a fluid can be imaged by adequately agitating the fluid environment, as demonstrated here in a simple proof-of-concept model. The model reveals that a small particle can be detected remotely without relying on any slow deformation or translation relative to the sensors, as commonly assumed in past models of hydrodynamic imaging. Instead, the mechanism proposed here relies on the edge of a viscous boundary layer on a moving surface to generate sufficient strain around a non-deforming particle, which could be a viscous droplet or air bubble with sufficient surface tension. It is this relative rigidity of the particle that importantly distorts the surrounding flow. A novel feature of generating impulsive or oscillatory motion is that the signal from the particle peaks at an intermediate time after bursting into motion or at an intermediate frequency of oscillation, the exact optimum depending on the distance to the particle. Thus the frequency or time scale could be tuned to systematically vary the region of maximal agitation and thereby scan for particles at different distances, contrary to the same proximal region that would be agitated around a body cruising at constant velocity. The predicted signals are detectable in theory by fish, copepods and artificial devices, though the signals may differ significantly in reality due to numerous simplifications made in the model. Therefore, the next steps in research are to consider the process of sensing particles in more complex flow fields around bodies with different shapes and dynamics. This raises the challenge of filtering useful signals from noisy background flow fields. The corresponding problem in acoustic fields has been a subject of great interest to sensory physiologists and ecologists, e.g., in understanding how crickets and frogs find their mate in a plethora of mating calls^39^. One solution to be considered would be Wehner’s explanation of “matched filters”^40^, which found acceptance lately in the book by von der Emde and Warrant^41^. Further research is needed to explore possible strategies for various organisms and potentially machines to enhance their range of perception.

## Methods

We first outline the method for predicting the motion of a force-free and torque-free sphere centered at ${{\bf{x}}}^{\ast }$ in a background flow ${\bf{u}}$. The sphere’s translational velocity ${{\bf{u}}}_{p}$, angular velocity ${{\bf{w}}}_{p}$, and stresslet ${\bf{S}}$ exerted by the flow are related to the force ${\bf{F}}$, torque ${\bf{T}}$, and strain-rate ${\bf{E}}$ evaluated at ${{\bf{x}}}^{\ast }$ according to Faxen’s laws^42^

22 $${\bf{F}}=6\pi \mu a({\bf{u}}({{\bf{x}}}^{\ast })+\frac{{a}^{2}}{6}{\nabla }^{2}{\bf{u}}({{\bf{x}}}^{\ast })-{{\bf{u}}}_{p}),$$

23 $${\bf{T}}=8\pi \mu {a}^{3}(\frac{1}{2}\nabla \times {\bf{u}}({{\bf{x}}}^{\ast })-{{\bf{w}}}_{p}),$$

24 $${\bf{S}}=\frac{20}{3}\pi \mu {a}^{3}(1+\frac{{a}^{2}}{10}{\nabla }^{2}){\bf{E}}.$$

Equations (4–6) are obtained by setting ${\bf{F}}={\bf{T}}=0$ and using Eq. (1) as the background flow near an impulsive plate. Similarly, Eqs. (14–17) are obtained using Eq. (11) as the background flow near an oscillatory plate.

The effect of the stresslet exerted by the sphere is analyzed as follows. For a general stresslet ${S}_{jk}$ located at ${\bf{y}}$, the flow velocity ${u}_{i}^{S}({\bf{x}})$ and pressure ${p}^{S}({\bf{x}})$ are given by^43^

25 $${u}_{i}^{S}({\bf{x}})={T}_{ijk}({\bf{x}},{\bf{y}}){S}_{jk},\,{p}^{S}({\bf{x}})={\Pi }_{jk}({\bf{x}},{\bf{y}}){S}_{jk},$$

where the indices *j* and *k* are summed from 1 to 3 and

26 $${T}_{ijk}({\bf{x}},{\bf{y}})=\frac{3}{8\pi \mu }\frac{({x}_{i}-{y}_{i})({x}_{j}-{y}_{j})({x}_{k}-{y}_{k})}{|{\bf{x}}-{\bf{y}}{|}^{5}},$$

27 $${\Pi }_{jk}({\bf{x}},{\bf{y}})=\frac{1}{4\pi }(-\frac{{\delta }_{jk}}{|{\bf{x}}-{\bf{y}}{|}^{3}}+\frac{3({x}_{j}-{y}_{j})({x}_{k}-{y}_{k})}{|{\bf{x}}-{\bf{y}}{|}^{5}}).$$

In our model, the non-zero components of the stresslet ${S}_{13}$ and ${S}_{31}$ are set equal to the stresslet strength *S*.

The image system for imposing the no-slip condition on the plate can be obtained using classical methods based on Fourier transforms^44^ or the Lorentz reciprocal theorem^43,45^. Here we adopt an arguably simpler method using a scalar harmonic Papkovich-Neuber potential as developed relatively recently^26^. The disturbance flow satisfying no slip on the plate is represented by

28 $${u}_{i}({\bf{x}})={T}_{ijk}({\bf{x}},{\bf{y}}){S}_{jk}-{T}_{ijk}({\bf{x}},{{\bf{y}}}^{{\bf{I}}}){S}_{jk}^{I}-({x}_{3}\frac{\partial \phi }{\partial {x}_{i}}-{\delta }_{i3}\phi ),$$

which consists of three parts: the original stresslet ${S}_{jk}$ at the centre of the sphere ${\bf{y}}=(0,0,h)$, an image stresslet ${S}_{jk}^{I}=-\,{S}_{jk}$ at ${{\bf{y}}}^{I}=(0,0,-\,h)$ corresponding to the reflection of the centre of the sphere in the plane of the plate, and the remaining terms contributing from a Papkovich-Neuber potential

29 $$\phi ={S}_{jj}^{I}\frac{\partial }{\partial {y}_{3}}\frac{1}{4\pi \mu |{\bf{x}}-{{\bf{y}}}^{I}|}+h{S}_{jk}^{I}\frac{{\partial }^{2}}{\partial {y}_{j}\partial {y}_{k}}\frac{1}{4\pi \mu |{\bf{x}}-{{\bf{y}}}^{I}|}.$$

The first term vanishes because the stresslet is traceless. The remaining contribution to the potential corresponds to a source quadrupole at ${{\bf{y}}}^{I}$. The disturbance flow adjusts the particle speed in Eq. (4) by a small correction of order ${\alpha }^{3}$ which is neglected for simplicity. The disturbance in pressure is given by

30 $$p({\bf{x}})={\Pi }_{jk}({\bf{x}},{\bf{y}}){S}_{jk}-{\Pi }_{jk}({\bf{x}},{{\bf{y}}}^{{\bf{I}}}){S}_{jk}^{I}+2\mu \frac{\partial \phi }{\partial {x}_{3}},$$

which consists of three parts again. The original and image stresslets cancel each other out at ${x}_{3}=0$, leaving with only the source-quadrupole potential to contribute to the pressure on the plate. Equations (28–30) are evaluated at ${x}_{3}=0$ to obtain the normal and shear stresses on the plate given in Eqs. (7–9).

## Supplementary information

Supplementary Movie 1.

SupplementaryMovie 2.

Supplementary Movie Legends.
