# Supplementary material for: Active hydrodynamic imaging of a rigid spherical particle
Source: Sci Rep. 2020 Feb 14;10:2665. doi: 10.1038/s41598-020-58880-0 (PMC7021710; doi:10.1038/s41598-020-58880-0)
Supplement: Supplementary file 3 — Supplementary Movie Legends. [file 41598_2020_58880_MOESM3_ESM.pdf]

Active hydrodynamic imaging of a rigid spherical particle

Daisuke Takagi and J. Rudi Strickler

Supplementary Information

Supplementary Movie S1. A sphere moves in response to an impulsively started plate and induces pressure changes on the plate. The sphere is centered at a distance four times its radius from the plate. The time is dimensionless as defined in the main text.

Supplementary Movie S2. A sphere moves in response to an oscillatory plate and induces pressure changes on the plate as shown for two periodic cycles. The sphere is centered at a distance four times its radius from the plate. The Stokes boundary layer thickness is set equal to the distance between the sphere and the plate.
